# Advances in Therapeutic Options for Pulmonary and Sleep Disorders in Mucopolysaccharidosis (MPS) Patients: A Narrative Review

**DOI:** 10.3390/arm94030041

**Published:** 2026-06-22

**Authors:** Bimaje Akpa

**Affiliations:** Division of Pulmonary, Critical Care and Sleep Medicine, Department of Medicine, University of Minnesota Medical School, Minneapolis, MN 55455, USA; akpa0012@umn.edu

**Keywords:** mucopolysaccharidosis, pulmonary disorders, sleep disorders, advances in treatment

## Abstract

**Highlights:**

**What are the main findings?**

- The use of enzyme replacement therapy (ERT) and hematopoietic stem cell transplantation (HSCT) have fundamentally altered the disease course by mitigating systemic glycosaminoglycan (GAG) buildup, with emerging gene therapies showing significant promise for respiratory optimization.
- MPS patients have a relatively high prevalence of sleep disordered breathing and face significantly higher risk during surgeries due to macroglossia, airway obstruction, cervical spine instability, and restrictive lung disease.

**What are the implications of the main findings?**

- ERT and HSCT are far more effective before irreversible tissue damage occurs, the inclusion of MPS in newborn screening programs and early detection are strongly recommended.
- Optimal management requires highly collaborative care among pulmonologists, otolaryngologists, and sleep specialists to mitigate peri-operative risks and deploy positive airway pressure devices when needed.

**Abstract:**

Mucopolysaccharidosis (MPS) are a group of inherited lysosomal storage genetic disorders that affect the body’s ability to break down glycosaminoglycans (GAGs) due to the deficiency of required enzymes. This leads to depositions of these GAGs in various tissues and organs resulting in multi-systemic manifestations including pulmonary and sleep related issues. In recent years, there have been significant advancements in therapeutic options and supportive management which have led to the overall improvement in respiratory care, culminating in improved quality of life for MPS patients. Management of pulmonary and sleep disorders in mucopolysaccharidosis requires a multidisciplinary approach due to the multi-systemic affectation of the genetic disorders. Therapeutic options such as enzyme replacement therapy (ERT) and hematopoietic stem cell transplantation (HSCT) have yielded varying success in mitigating respiratory complications. Emerging treatments such as gene therapies have shown exciting and promising results thus far. Supportive therapies such as airway clearance, regular vaccination and use of positive airway pressure devices are also essential. Pre-operative airway and anesthesia planning is critical to mitigate peri-operative and post-operative complications. Early diagnosis, close monitoring and a patient focused individualized approach are essential for respiratory optimization and overall improvement in clinical outcomes. This review article aims to discuss these advancements in a comprehensive format, making it accessible to medical providers who care for this subset of patients.

## 1. Introduction

Mucopolysaccharidosis (MPS), also known as lysosomal storage diseases (LSD), is characterized by the accumulation of GAGs in tissues and organs caused by genetic disorders (See Table 1), leading to deficiency in enzymes required for their degradation [1,2]. GAGs are long chains of polysaccharides found in cells and tissues which are required in cellular functional processes such as cellular signaling and adhesion [1]. Deposition of these GAGs in the lysosomes of cells is the primary cause of MPS resulting in multi-systemic manifestations including respiratory, airway and sleep-related disorders [3].

This accumulation manifests as a spectrum of clinical features, including skeletal dysplasia, cardiac involvement, and neurological impairment, with pulmonary and sleep disorders emerging as critical complications that significantly impact morbidity and mortality. In MPS, GAG deposition in airway tissues, the chest wall, and lungs results in upper and lower airway obstruction, restrictive pulmonary disease, and obstructive sleep apnea (OSA), often presenting early in life and exacerbating with disease progression. These multi-systemic issues are complicated by structural abnormalities like macroglossia, adenotonsillar hypertrophy, and chest wall deformities, which impair ventilation and sleep quality, ultimately affecting overall survival and health-related quality of life.

## 2. Methods

### 2.1. Search Strategy

A systematic search of major medical databases (PubMed, Embase, Cochrane Library) was conducted for articles published between January 1960 and October 2025 to identify studies, and review papers on diagnosis and management of pulmonary and sleep disorders in MPS disease. Keywords (e.g., ‘mucopolysaccharidosis’, ‘pulmonary disorders’, ‘sleep disorders’, ‘advances in treatment’) were combined using Boolean operators. Titles and abstracts were screened, followed by full-text review to ensure focus on therapeutic options for pulmonary and sleep disorders in MPS disease.

Initial database searching identified 400 records. After duplicate removal and relevance-based filtering, 200 records were selected. Of these, 98 papers were excluded based on eligibility criteria (below), resulting in 102 papers being included in the final synthesis.

Eligibility criteria included:

MPS Focus: Does the paper specifically address mucopolysaccharidosis?

Pulmonary or Sleep Content: Does the paper discuss pulmonary, airway, or sleep disorders in MPS?

Management Discussion: Does the paper cover management, treatment, or interventions for pulmonary or sleep disorders in MPS?

Clinical Relevance: Is the content clinically relevant? (e.g., retrospective/prospective cohort, case-control, cross-sectional, case series, random controlled trials, cross-sectional survey with focused reporting on therapeutic options in management of respiratory and sleep disorders in MPS patients) Does it measure clinical outcomes like endurance, pulmonary function and severity of sleep disordered breathing?

Outcome Reporting: Does the paper report outcomes or efficacy of management strategies?

Review Type: Is the paper a review, guideline, or consensus statement?

### 2.2. Relevant Section

**Physiology of pulmonary and sleep complications in MPS**

**Figure 1:**
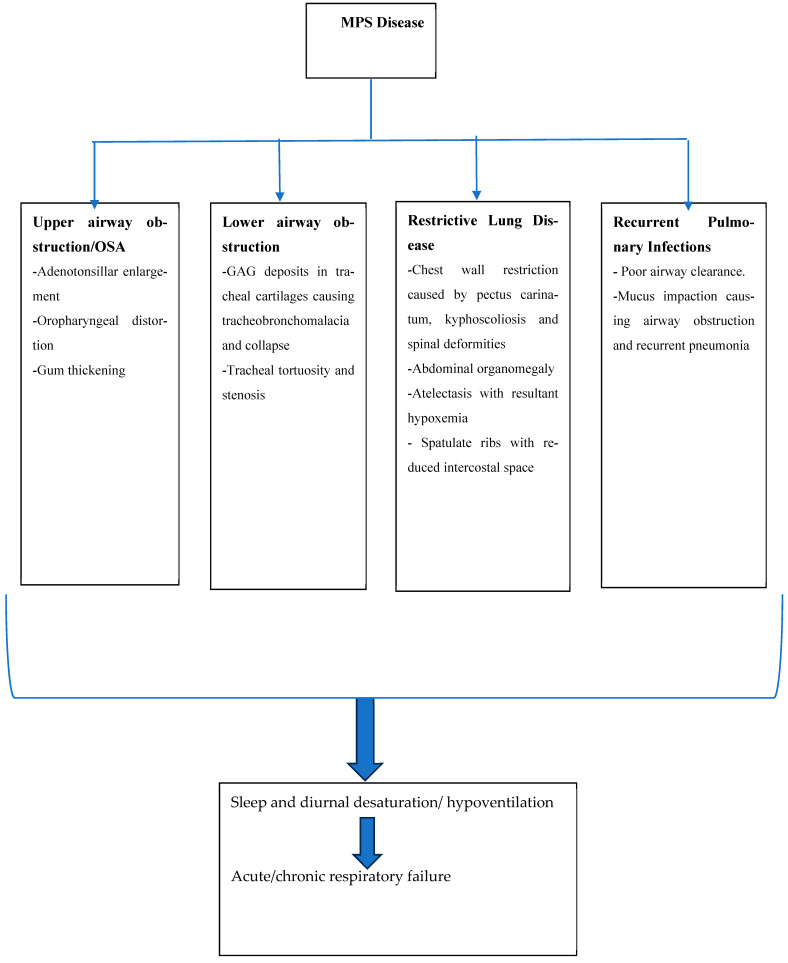
Pathophysiology of respiratory and sleep disorders in MPS.

Sleep and pulmonary involvement are usually progressive and have varying manifestation depending on the specific type of MPS. The various mechanisms (see Figure 1) in which MPS causes sleep disordered breathing and pulmonary issues include:

**Tracheal collapse and airway obstruction**

Pulmonary involvement in MPS is characterized by upper airway obstruction due to GAG deposition causing adenotonsillar hypertrophy, macroglossia, and tracheal narrowing. Functional and structural changes occur in the upper and lower airway due to deposition of GAGs [4,5,6,7]. These results in tonsillar and adenoidal enlargement, macroglossia and tracheobronchomalacia. Resultant oropharyngeal, supraglottic airway collapse and tracheal changes (tracheal stenosis and tortuosity) can also lead to airway obstruction and collapse respectively [8,9,10,11,12]. In MPS I and II, these features manifest early, with neonatal respiratory distress in 32% of MPS II cases [13] and progressive multilevel obstruction in MPS II contributing to mortality via tracheal collapse. In adults, airway tortuosity and narrowing persist, quantified by the Salford MPS Airway Score correlating poorly with BMI [9].

**Sleep disordered breathing (SDB)**

Physiologic changes that occur during sleep lead to pharyngeal muscular relaxation leading to worsening airway obstruction. This causes obstructive sleep apnea, sleep hypoxemia and hypoventilation. Untreated significant SDB may lead to neuro-cardiac complications such as strokes, hypertension, coronary artery disease and pulmonary hypertension. SDB is highly prevalent in MPS patients with some studies reporting about 80% [2,6,14,15]. The MPS types most affected include I and II [4,15,16,17]. Pretreatment rates of OSA in a particular study were 81%, with a mean apnea-hypopnea index (AHI) of 10.4 events/hour overall, escalating to 16.6 in MPS I where 75% experienced moderate-to-severe disease [18]. Almost all MPS patients have adenotonsillar hypertrophy, but the cause of OSA is usually multifactorial. Other causes of sleep disordered breathing include GAG deposition in upper airway, crowded retroglossal and retropalatal spaces, small chest wall, macroglossia, and short neck [19,20].

**Restrictive lung disease**

Pulmonary involvement in MPS is characterized by restrictive lung disease from skeletal deformities and reduced chest wall compliance, leading to recurrent infections and impaired gas exchange. Restrictive patterns predominate, with reduced forced vital capacity linked to thoracic kyphosis in MPS I [21]. Hurler Chest wall restriction caused by kyphoscoliosis, broad spatulate ribs with reduced intercostal space, pectus carinatum, spinal disorders are common in MPS patients. There can be complicated with ventilatory failure due to reduced lung volumes. Abdominal enlargement due to hepatosplenomegaly can cause diaphragmatic dysfunction due to compressive effects [2,7,16,22].

### 2.3. Disease-Targeting Therapies

Enzyme replacement therapy (ERT): ERT can be an effective therapeutic option in MPS types 1, II, IVA, VI and VII [23,24,25,26,27] (See Table 2). It involves intermittent administration of intravenous recumbent enzymes to address the deficiency of lysosomal enzymes in these patients [25,26,28]. This aims to reduce the GAG burden and accumulation in the tissues of the respiratory tract. ERT has shown varying levels of benefits in pulmonary function, sleep disordered breathing and airway obstruction [24,27,29,30]. ERT can cause patients to develop anti-drug antibodies (ADA) in response to treatment. Some of these ADAs can be inhibitory and negatively affect the overall efficacy of ERT [18]. A study showed that ERT improved obstructive sleep apnea in MPS patients with low levels of inhibitory antibodies [18]. Another study showed no positive effect of ERT on OSA in MPS II [31]. The effect of ERT on reducing adenotonsillar enlargement was negligible [31,32,33]. Overall, there is a paucity of data available on the long-term effect of ERT on OSA in MPS patients.

Spirometry evaluations in MPS usually involve measurements of forced expiratory volume in 1 s (FEV1) and forced vital capacity (FVC) in clinical and research settings [16,17,34,35]. The 6 min-walk test (6MWT) is the most common respiratory parameter used to evaluate endurance in MPS patients [24,25,36,37,38]. ERT also has varied and limited success in improving lung volumes, usually reaching a plateau within 2 years of starting therapy [39,40]. Several studies have shown short-term improvement in endurance after ERT, but long-term effects are limited [23,24,27,32,41,42]. ERT with laronidase (100 U/kg weekly) improves pulmonary function in MPS I, increasing forced vital capacity by 11% and 6-min walk distance by 38 m over 26 weeks, alongside urinary GAG reductions from 63.4 mg/mmol creatinine baseline to 16.3 mg/mmol at 24 months [43].

In MPS I and IVA, ERT has shown some improvements in these measures up to 5%, usually limited to the first year of treatment [44]. In other MPS types, there was no clinically significant improvement [24,45]. In prospective, longitudinal studies, the FVC and FEV1 overall changes in the long term were limited to less than 20% [28,31,41,46]. Galsulfase ERT has been recommended as the first line treatment therapy for MPS VI for over 20 years. In a recent 15 year-analytic clinical surveillance program for MPS VI, ERT-treated patients demonstrated persistent decrease in urinary GAG levels with associated improvements in pulmonary function and endurance over a prolonged follow-up timeframe [47]. Harmatz and colleagues demonstrated a sustained improvement in walking distance for an extended period in an open-label extension study in MPS VI patients [24]. In MPS II, idursulfase ERT showed limited improvement in pulmonary function, with function tests unhelpful due to disabilities, though growth dynamics indicate anabolic effects in <50% mild cases [48]. Respiratory dysfunction in MPS is usually multi-factorial: GAG accumulation in the soft tissues of the pharyngeal and upper airway with resultant obstruction; tracheobronchomalacia and stenosis causing lower airway narrowing and collapse respectively; and restrictive thoracic disorders due to chest wall, rib and spinal deformities. Since the major principle for the therapeutic efficacy of ERT is based on the reduction in GAG deposition, therefore it appears to be relatively more effective on soft tissues and upper airway obstruction [5,6,9,16,20]. Conventional ERT is largely ineffective in treating skeletal and cartilage abnormalities related to MPS due to poor circulation in these tissues which makes it less effective in chest wall and tracheal deformities. Additionally, conventional ERT has a short circulatory half-life of the infused recombinant enzymes [49,50]. In patients with MPS I and II, most of the therapeutic enzyme is rapidly cleared from the bloodstream and becomes virtually undetectable in plasma just a few hours post-infusion [51]. This rapid clearance occurs because the enzymes are aggressively captured by carbohydrate-recognizing receptors, such as mannose 6-phosphate (M6P) receptors which preferentially shunt the protein into highly vascularized visceral organs like the liver, spleen and less so with hypovascular bones and cartilage tissues [52]. The therapeutic window of systemic enzyme availability is simply too narrow to permit meaningful deep-tissue penetration. As a result of these factors, structural changes in cartilages and bony structures are usually less reversible [5,12,26,31,41,49]. Pre-clinical studies showed that using a long-circulating form of enzymes (Pert-GUS) significantly improved bone lesions due to enhanced circulation [53]. Similarly, due to the inability of ERT to cross the blood–brain barrier (BBB), it is ineffective in treating neurological manifestations due to MPS. A recent therapy for MPS II—Hunter syndrome (Pabinafusp Alfa) using fusion of transferrin receptor antibody with idursulfase enzyme improves delivery across the BBB therefore improving efficacy for treatment of neurological symptoms [54].

In summary, these studies show that ERT significantly improves lung function with endurance, reduces AHI, and improves sleep quality. Early initiation of ERT is crucial, as it is most effective at reducing GAG storage in soft tissues and stabilizing pulmonary and sleep disorders before irreversible anatomical damage occurs.

Hematopoietic Stem Cell Transplantation (HSCT): HSCT is a proven long-term treatment for certain MPS types including MPS I, MPS II, MPS IVA, MPS VI, and MPS VII [41,50,55,56,57,58] (See Table 2). It involves transplanting matched donor stem cells to provide the deficient enzyme. The efficacy of HSCT is dependent on factors such as patient age, MPS type, donor selection and overall functional status. The age at which HSCT is performed affects the overall efficacy of treatment with outcomes vary by timing, with early HSCT (<2 years) improving survival [55,57,59]. Several studies have shown that cord blood transplantation usually is more effective and has a relatively lower rate of draft versus host disease (GVHD) compared to traditional bone marrow transplantation (BMT) [60,61]. The latter option may be more suitable for adult and older MPS patients due to less incidence of GVHD. Unlike ERT where the infused deficient enzymes are unable to cross the BBB, donor stem cells in HSCT can cross the barrier, hence providing neurologic improvements. Initiating HSCT before the age of two is critical in MPS patients to halt the progression to severe respiratory impairment, such as restrictive lung disease and upper airway obstruction [62,63]. Because irreversible tissue and organ damage often takes hold before age 3, robust Newborn Screening Programs and early diagnosis are essential for timely, life-preserving intervention [64].

HSCT has shown promise in the treatment of respiratory and airway issues, especially in young patients with MPS I. Walker et al. revealed a significant reduction in the incidence of airway complications in MPS I patients treated with HSCT compared to ERT (14% vs. 57%) [65]. A retrospective study reported no intubation failures in MPS I patients previously managed with HSCT [56]. Another study showed improved respiratory outcomes in a small number of MPS IH patients [63]. These findings suggest that there is clinically significant improvement in airway management, intubation success, as well as the overall safety of anesthesia.

Yabe and colleagues reported cessation of orthopnea, snoring and improved respiratory function in four MPS IVA patients after HSCT [66]. Careful selection for MPS IVA patients is necessary as they have severely narrowed airway due to tracheobronchial distortion which leads to difficult intubation, peri-intubation respiratory complications and reduced success of ventilator liberation overall [67]. In the largest non-pharmaceutically supported retrospective study, pediatric MPS I patients who received either ERT or HSCT, serial spirometric evaluations demonstrated a universal degree of restrictive lung function irrespective of the therapeutic intervention. During the follow up, the level of restrictive lung disease stabilized in 67% of post HSCT and 57% in post ERT in MPS I patients [34]. There has been varying success in improvement in sleep disordered breathing, airway obstruction and lung function improved lung function in other MPS types. Use of HSCT in MPS patients carries significant risks, primarily driven by myeloablative conditioning [68]. The required intense chemoradiation can lead to severe regimen-related toxicities, including profound, often permanent infertility [69]. Furthermore, these myeloablative therapies frequently cause endocrine disruptions that result in growth retardation. Because these considerable risks can adversely impact long-term quality of life and survivorship, ERT may be utilized instead particularly with attenuated disease and in MPS patients with significant neurological involvement [70].

In summary, the early use of HSCT can diminish upper airway obstruction and reduce OSA, but late complications may still require additional treatments such as surgeries. Phenotypic variability within the same MPS type—often driven by the extent of residual enzyme activity and level/type of airway structural damage—dictates both the severity of airway narrowing and a patient’s response to therapies like ERT and HSCT [71,72]. Patients with attenuated phenotypes typically exhibit milder airway narrowing and slower disease progression, allowing treatments to better stabilize or moderately improve pulmonary function and sleep-disordered breathing. Conversely, those with severe phenotypes develop profound, irreversible cartilage deformities and tracheobronchomalacia, causing limited response in respiratory function to these treatments [72,73].

The combination of HSCT and ERT has shown promise in some studies. as an optimal approach for conditions like Hurler syndrome (MPS I) [74,75]. ERT mitigates pre-transplant pulmonary symptoms, while HSCT introduces a continuous, endogenous source of enzymes. ERT is frequently administered during the wait for a matched donor to rapidly clear GAGs, alleviating airway obstruction and stabilizing pulmonary function so the patient is healthier going into chemotherapy and transplantation [75].

It is also theorized that pre-transplant ERT clearance of GAGs in bone marrow may aid stem cell engraftment, leading to higher overall survival rates [74].

HSCT and ERT exhibit significantly different cost-effectiveness profiles for MPS long term. ERT requires lifelong, repeated, and highly expensive intravenous infusions, which can cost up to USD 550,000 annually depending on the patient’s weight and the specific MPS type [2]. By contrast, HSCT functions as a one-time curative procedure with upfront costs ranging generally from USD 60,000 to 200,000 depending on the donor, conditioning regimen, and location. Because HSCT provides a continuous, internal supply of the deficient enzyme—it may represent a highly cost-effective relative to chronic, lifelong ERT for patients [70,76].

### 2.4. Surgical Interventions

Surgical interventions play a fundamental role in managing upper and lower airway obstruction in MPS patients (see Table 2).

**Adenotonsillectomy (AT)**: Adenotonsillectomy is the first line treatment for OSA in pediatric MPS patients and about half of this subset of patients undergo the surgery [77]. Few studies have reviewed the efficacy of AT and the outcomes are usually non-lasting likely due to the multifactorial pathogenesis, progressive nature of MPS disease as well as recurrence of adenoid hypertrophy. The recurrence rate of adenoid hypertrophy has been reported to be up to 56% versus 1.9% in healthy counterparts [78,79]. Lee et al. showed more than 50% reduction in AHI in about a quarter of patients (15 patients) [10].

**Tracheostomy**: Tracheostomy is a surgical intervention which can be feasible and helpful for isolated upper airway obstruction. Ten percent of MPS patients usually undergo tracheostomy for airway issues [80]. 47% of cases of MPS II patients were reported to undergo tracheostomy which was the most frequent subtype of MPS [80]. This underscores the fact that this subset of patients is at a higher risk of respiratory failure due to progressive airway obstruction secondary to GAG deposition. Patients may develop lower or multi-level airway obstruction which makes finding the right tracheostomy tube for a patient with MPS very challenging [80]. AT and other surgeries are usually unsuccessful for permanently alleviating airway obstruction [4,7,8,65]. It may be necessary to undergo tracheostomy when other surgeries have failed. Tracheostomy may be challenging in MPS patients due to peculiar anatomical features such as stiff short neck, low hanging cricoid ring, multi-level tracheal obstruction and large protruding mandible [80]. Airway obstruction may persist despite tracheostomy due to airway collapse distal to the tip of the tube [81].

**Tracheal resection and reconstruction**: This is applicable in cases when there are significant narrowing and collapse of a segment of the trachea for which tracheostomy may not be applicable. MPS IVA can have very significant tracheal narrowing, and this procedure can significantly improve pulmonary function and quality of life in this subset of patients [5,11,50,55,67,82]. Pizarro et al. [83] and Hack et al. [84] described tracheal resection via median sternotomy with post operative improvement in patient-reported outcomes and quality of life.

Kenth et al. evaluated sixteen patients with severe MPS IVA and radiological evidence of advanced airway obstruction who underwent tracheal resection with combined manubrial resection. Post operatively, there were significant spirometric improvements including a mean increase of 0.68 L in forced expiratory lung volume in 1s (FEV1) [85]. There were no reported long-term complications. These findings highlighted the significant improvements in patient-reported outcomes and overall quality of life.

**Tracheobronchial stents**: The use of airway stents have been established in adult malignant airway disease. Pediatric use of these stents has been sparse but slowly increasing. Airway stent placement is usually performed in the United States by experienced interventional pulmonologists and thoracic surgeons. The reported use of airway stents in MPS patients are sparse with only a handful of cases [86]. Stents are prone to accumulation of biofilms, mucus plugging, granulation formation and migration. Insertion and replacement of these stents are extremely challenging in MPS patients [86]. Stents may be very beneficial with short-term success as a bridge to other permanent solutions such as tracheal resection [86]. They are very feasible for palliative care given the short-term improvement in quality of life.

### 2.5. Supportive Therapies

Supportive therapies are crucial for optimizing respiratory function, endurance and overall wellbeing in MPS patients.

**Prevention of Anesthetic and post-operative complications**—As a result of the multi-systemic and complex nature of the disease, MPS patients usually require several procedures and surgical interventions. MPS patients carry the highest reported intra and peri-operative mortality because of airway, pulmonary, cardiac and neurologic issues [4,41,65,87,88]. MPS I, II, IV have the highest rates of airway complication due to the relatively high prevalence of adenotonsillar hypertrophy, copious thick secretions, macroglossia, facial dysmorphism and airway collapse due to laryngomalacia. The airway mucosa is very friable with easy bleeding. MPS I and IV may have unstable atlanto-axial joint, short neck and decreased mobility of the temporo-mandibular joint which makes intubation very difficult with increased complications [41,87,88,89].

A team approach for anesthesia is essential for pre-operative assessment for MPS patients. A pre-operative evaluation with an individualized plan is important to reduce these risks. Patients deemed to be high risk should have a pre-operative evaluation jointly by the anaesthetic, ENT and pulmonary team and risks of the airway intervention should be discussed across the benefits of surgery. A collective multidisciplinary approach is strongly recommended [65,88]. A thorough evaluation should be done by detailed history and where possible- nasal endoscopy, CT scans and PFTs should be obtained. Airway intervention risks can be based on previous anesthetics and holistic lip-to-lung assessments. Post-operative complications are reduced by staged extubation, optimal use of opiates, early physiotherapy and mucolytics [65,90]. Patients with a history of difficult intubations should be flagged on electronic medical records for easy identification. Awake flexible fiberoptic airway examination and bronchoscopic intubations may lead to lesser airway injury compared to standard video laryngoscopy [41,87]. 3D airway reconstruction, virtual endoscopy and the use of the Salford MPS airway score can also help mitigate airway intervention risks [9].

**Positive airway pressure devices**—There are several therapeutic options for the treatment of OSA and sleep disordered breathing in MPS patients including adenotonsillectomy, ERT, HSCT and tracheostomy. Given the non-invasive approach and the likely sustained efficacy with multi-level upper airway obstruction, CPAP is advantageous compared to the other options [14,18,20]. Tolerance may be an issue particularly with MPS patients due to behavioral issues [16,18,20].PAP mask tolerance may be affected by facial dysmorphism, large tongue and oropharyngeal changes [14,16,22]. Using a nasal mask may be better tolerable. Use of bi-level PAP devices may be needed if CPAP is unable to effectively treat nocturnal hypoventilation and desaturation. There is increasing use of 3D-printing to help with customized mask production for this sub-set of patients to improve adherence [91]. Close team working with long term ventilation team and respiratory physiotherapists, pulmonologists and sleep specialists is important to find the right CPAP equipment which fits the MPS patients [14].

**Insomnia**

Insomnia can lead to increased morbidity and reduced overall wellbeing of MPS patients [92,93,94]. It may be related to circadian misalignment due to involvement of the retina and central nervous system. Melatonin has been used to treat sleep disturbance with significant success in MPS patients [92]. It also has a superior side effect profile compared to other sleep aides such as benzodiazepine [92,94]. Benzodiazepines are better avoided due to underlying complex airway and sleep disordered breathing [95].

**Secretion Management and Airway Clearance Techniques**—Due to excessive and thick lung secretions, airway clearance techniques such as manual chest physiotherapy, chest wall oscillatory percussion devices and mechanical devices may be needed to improve secretion mobilization as well as clearance [65]. The use of nebulized mucolytics is also very helpful [65]. Inhaled corticosteroids, bronchodilators, nasal decongestants may be used to address airway inflammation and associated symptoms such as nasal congestion, postnasal drip, chest congestion and wheezing [7,41]. Mucolytics such as acetylcysteine, saline and hypertonic saline nebulization, steam inhalation are all other useful methods [96].

**Regular vaccination**—Vaccinations against respiratory syncytial virus (RSV), influenza, SARS-CoV-2 and pneumococcal are essential to reduce the risk of respiratory infections [97].

**Aggressive management of respiratory infection and pneumonia**—Timely diagnosis of respiratory infections and aggressive treatment is critical in preventing complications of respiratory failure [7].

**Pulmonary rehabilitation**—MPS patients with chest wall disease, spinal deformities and neuromuscular weakness should be referred to pulmonary rehab programs to help maximize respiratory muscular strength and functionality [98].

**Spinal Decompression Surgery**—All MPS patients undergoing anesthetic intervention should be treated as unstable spine avoiding any hyper extension. MPS IV patients have odontoid dysplasia and hypermobility of the spine [99]. MPS patients with critical spinal cord compression affecting respiratory muscular functionality should be referred for surgical decompression which may help improve pulmonary function [100].

**Importance of a multidisciplinary approach—The MPS Team**

The multi-systemic involvement, complex and progressive nature of the disease requires a dedicated multidisciplinary team including consultants in pulmonology, neurology, general pediatrics, genetics, otolaryngologist and anesthesia (see Table 3). An MPS program is needed for collaborative care across specialties to address the individualized needs of MPS patients and to maximize the quality of life and overall long-term health [101,102].

This narrative review is subject to several limitations, primarily stemming from the rarity of the disease which results in a reliance on small study populations rather than large prospective randomized controlled trials. The therapeutic landscape, including ERT and HSCT, presents challenges in evaluating long-term efficacy due to the progressive nature of airway obstruction and the potential for treatment benefits to wane over time. Finally, as a narrative review, this does not provide a systematic meta-analysis, making it difficult to firmly establish the impact of new therapies on disease progression.

## 3. Limitations

This review has several limitations. Studies encompass predominant case series and retrospective data which are prone to selection bias, with small samples limiting generalizability; polysomnography metrics vary (e.g., AHI vs. desaturation index), hindering direct comparisons, and few address adult patients. Mechanistic insights are sparse beyond GAG deposition.

## 4. Gaps and Future Directions

Future clinical studies should conduct prospective, multicenter trials in exact MPS populations (e.g., severe vs. attenuated) using synchronized polysomnography and pulmonary function tests and modalities to quantify long-term intervention effects. Methodological advances like 3D airway imaging could strengthen subtype comparisons, while targeted research in underrepresented adults and low-resource contexts would address access biases. There are also promising alternative therapeutic options which show high promises including gene therapy and genetic engineering using autologous B cells, to continuously secrete and replace missing enzymes.

## 5. Conclusions

Therapeutic advances in the management of respiratory and sleep disorders have improved clinical outcomes in MPS diseases. The integration of early-stage ERT, improved HSCT techniques, and early surgical intervention for airway obstruction have significantly altered the disease course, allowing patients to live longer and with better quality of life. However, management of advanced airway structural changes remains a critical challenge, requiring a multidisciplinary, long-term approach to minimize mortality and improve quality of life.

## Figures and Tables

**Table 1:** MPS types and enzyme deficiency.

MPS Type	Syndrome Name	Enzyme Deficiency	OMIM
1 (H, HS, S)	Hurler, Hurler-Scheie, Scheie	Dermatan sulphate, Heparan sulphate	607014, 607015, 607016
II	Hunter	Dermatan sulphate, Heparan sulphate	309900
III (A, B, C, D)	Sanfilippo	Heparan sulphate	252900, 252920, 252930, 252940
IVa	Morquio	Keratan Sulphate, Chondroitin sulphate	253000
VI	Maroteaux-Lamy	Dermatan sulphate, Chondroitin sulphate	253200
II	Sly	Dermatan sulphate, Heparan sulphate, Chondroitin sulphate	253220
IX	Natowicz	Hyaluronidase	601492
X	MPS10	Mutation in gene ARSK	619698
S Plus Syndrome	MPSPS	Mutation in the VPS33A gene	617303

**Table 2:** Types of pulmonary manifestations in MPS with management strategies.

Pulmonary Manifestation	Description	MPS Types Involved	Management Strategies
Upper airway obstruction	Adenotonsillar enlargement, oropharyngeal distortion, leading to sleep disordered breathing. Abnormal teeth protrusion, mouth opening, high Mallampati class affects airway.	MPS I, II, IVA, VI, VII Minimal involvement in MPS III	Adenotonsillectomy, nasal steroid (more effective in young patients), tracheostomy (in recalcitrant cases), ERT, HSCT
Lower Airway Obstruction	GAG accumulation in tracheal cartilages causing collapse and tracheobronchomalacia. Tracheal stenosis and tortuosity MPS IVA has a peculiarity for severely narrowed airways due to tracheobronchial distortion.	MPS I, 11, IVA Minimal involvement in MPS III and other subtypes of MPS IV	PAP therapy particularly at night, use of tracheal and bronchial stents in very selected patients, inhaled steroids (airway inflammation), ERT/HSCT (less effective compared to upper airway obstruction), tracheostomy (for severe cases)
Restrictive Lung Disease	Chest wall restriction caused by pectus carinatum, broad spatulate ribs, reduced intercostal space, kyphoscoliosis and spinal deformities. Reduced lung volumes due to abdominal organomegaly. Atelectasis with resultant hypoxemia	MPS IV, VI Minimal involvement in MPS III	Spinal and chest wall surgeries (if applicable), ERT, HSCT (less effective for chest wall disorders compared to upper airway obstruction).
Sleep Disordered Breathing	Obstructive sleep apnea (OSA) and sleep hypoxemia/hypoventilation	MPS I, II	Weight loss, adenotonsillectomy, CPAP or Bilevel PAP devices, supplemental oxygen (may worsen hypoventilation), ERT, HSCT. Tracheostomy (in severe cases)
Recurrent Pulmonary Infection	Poor airway clearance causing upper respiratory infections, bronchitis and pneumonia. Reduced immunity.	All MPS types	Appropriately recommended vaccinations, use of mucolytics and cough assistance devices and maneuvers.

**Table 3:** MPS team members and roles.

MPS Team Member	Role in MPS Management
Pulmonologist	Provide respiratory care to MPS patients which may include positive airway pressure devices, airway clearance and cough assist devices, prescription of medications to address airway inflammation and help with secretion mobilization. Assessment and monitoring of lung function.
Otolaryngologist	Addressing complicated airway obstruction, performing adenotonsillectomy for sleep disordered breathing, airway planning prior to surgical interventions, management of recurrent otitis media and hearing issues
Anesthesiologist	Pre- and Peri-operative airway assessment plans and management
Neurologist	Evaluation and management of neurological complications, including and nerve impingement affecting overall mobility and functionality
Orthopedic/Spinal Surgeon	Management of skeletal deformities, chest wall and restrictive thoracic disorders, addressing spinal cord compression to improve functionality
Clinical Geneticist	Diagnosis of MPS type, understanding disease progression and implications for treatment
Cardiologist	Optimizing cardiac health to prevent complications particularly during vulnerable periods such as during anesthesia for surgical interventions

## Data Availability

Data sharing is not applicable to this article as no datasets were generated or analyzed during the current study.

## Abbreviations

MPS	mucopolysaccharidosis
GAG	Glycosaminoglycans
ERT	enzyme replacement therapy
HSCT	hematopoietic stem cell therapy
OSA	obstructive sleep apnea
CPAP	continuous positive airway pressure
PAP	positive airway pressure
FEV1	forced vital capacity in first second
FVC	forced vital capacity
6 MWT	6-min walk test
BMT	bone marrow transplant
AT	adenotonsillectomy
LSD	lysosomal storage disease
AHI	apnea hypopnea index
SDB	sleep disordered breathing
